# Influence of methylsulfonylmethane on markers of exercise recovery and performance in healthy men: a pilot study

**DOI:** 10.1186/1550-2783-9-S1-P13

**Published:** 2012-11-19

**Authors:** Douglas S Kalman, Samantha Feldman, Andrew R Scheinberg, Diane R Krieger, Richard J Bloomer

**Affiliations:** 1 Miami Research Associates, Nutrition/Endocrinology Department, Miami, FL 33143, USA; 2 The University of Memphis, Cardiorespiratory/Metabolic Laboratory, Memphis, TN 38152, USA

## Background

Methylsulfonylmethane (MSM) has been reported to provide anti-inflammatory and antioxidant effects in both animal and man. Strenuous resistance exercise has the potential to induce both inflammation and oxidative stress. Using a pilot (proof of concept) study design, we determined the influence of MSM on markers of exercise recovery and performance in healthy men.

## Methods

Eight, moderately exercise-trained men (27.1±6.9 yrs) were randomly assigned to ingest MSM (OptiMSM™) at either 1.5 grams per day or 3.0 grams per day for 30 days (28 days before and 2 days following exercise). Before and after the 28 day intervention period, subjects performed 18 sets of knee extension exercise in an attempt to induce muscle damage (and to be used partly as a measure of exercise performance). Sets 1-15 were performed at a predetermined weight for 10 repetitions each, while sets 16-18 were performed to muscular failure. Muscle soreness (using a 5-point Likert scale), fatigue (using the fatigue-inertia subset of the Profile of Mood States), blood antioxidant status (glutathione and Trolox Equivalent Antioxidant Capacity [TEAC]), and blood homocysteine were measured before and after exercise, pre and post intervention. Exercise performance (total work performed during sets 16-18 of knee extension testing) was also measured pre and post intervention.

## Results

Muscle soreness increased following exercise and a trend was noted for a reduction in muscle soreness with 3.0 grams versus 1.5 grams of MSM (p=0.080), with a 1.0 point difference between dosages. Fatigue was slightly reduced with MSM (p=0.073 with 3.0 grams; p=0.087 for both dosages combined). TEAC increased significantly following exercise with 3.0 grams of MSM (p=0.035), while homocysteine decreased following exercise for both dosages combined (p=0.007). No significant effects were noted for glutathione or total work performed during knee extension testing (p>0.05).

## Conclusion

MSM, especially when provided at 3.0 grams per day, may favorably influence selected markers of exercise recovery. More studies are indicated to extend the work and findings of this pilot trial.

